# The Impact of Frequent Glucose Monitoring on the Prevalence of Complications Among Patients With Diabetes in Saudi Arabia

**DOI:** 10.7759/cureus.39796

**Published:** 2023-05-31

**Authors:** Maram T Alkhatieb, Khalid M Aljehani, Hussain A Alkhalifah, Nawaf S Alghamdi, Abdulrahman S Almaghrabi, Bader B Alqarni, Abdulrahman Y Alzahrani

**Affiliations:** 1 Department of Surgery, King Abdulaziz University Faculty of Medicine, Jeddah, SAU; 2 Faculty of Medicine, King Abdulaziz University, Jeddah, SAU

**Keywords:** cross-sectional study, saudi arabia, diabetes mellitus complications, continuous glucose monitoring, blood glucose level, glucose monitoring

## Abstract

Background: Diabetes mellitus (DM), including type 1 diabetes (T1D) and type 2 diabetes (T2D), affects the absorption of glucose from the blood. DM has serious complications that can be prevented by adequate knowledge of the disease and its complications, a healthy lifestyle, a modified diet, and regular glucose monitoring. Hence, this study aimed to assess the effects of frequent glucose monitoring on the occurrence of DM complications.

Methods: This cross-sectional study was performed at King Abdulaziz University Hospital between June and December 2022 and included patients with T1D or T2D. After consent, participants who agreed to join filled out an online questionnaire that was used to acquire information, such as demographic data, type of diabetes, blood glucose monitoring, and diabetic complications.

Results: A total of 206 diabetic patients participated in this study, with a mean age of 41.2±19.37, with 53.4% having T1D. Most participants monitored their glucose levels (85.4%), and the majority (65.3%) monitored them once or more daily. Patients who monitored their glucose levels more frequently had significantly fewer complications (p = 0.002). Continuous glucose monitoring (CGM) was the best monitoring method, as it demonstrated the lowest rate of complications compared to other methods (p = 0.002).

Conclusions: Frequent glucose monitoring and the use of CGM devices were associated with a decreased number of DM complications. Thus, we recommend that physicians encourage patients to perform CGM as it helps increase the frequency of monitoring.

## Introduction

Diabetes mellitus (DM) is defined as a disorder that affects the mechanism of glucose absorption in the blood and is classified into different categories, including type 1 diabetes (T1D) and type 2 diabetes (T2D) [1]. DM has quadrupled globally over the past 30 years and is now the ninth leading cause of mortality worldwide. One in 11 adolescents develops DM, with T2D accounting for 90% of cases [2]. In Saudi Arabia, the prevalence of diabetes has increased by 10 folds over the last three decades [3]. Moreover, the World Health Organization predicted that the number of patients with DM will increase from 89,000 in 2000 to 2,523,000 in 2030 [4]. Only a few studies have explored the prevalence of diabetic complications in the Saudi Arabian population, one of which reported that the prevalence of neuropathy in 552 diabetic patients was 19.9% [5]. The most important requirement for a patient diagnosed with DM is regular monitoring of blood sugar levels [6]. Self-monitoring of blood glucose (SMBG), continuous glucose monitoring (CGM), and regular glucometer testing have all been found to be essential for enhancing glycemic management and preventing microvascular complications, such as neuropathy, nephropathy, and retinopathy, as well as macrovascular complications, such as cardiovascular comorbidities [6-8].

A questionnaire-based descriptive study published in 2016 showed that among 630 patients with DM visiting the Diabetic Clinical Center at Sampa Government Hospital, Ghana, 378 (60%) did not have adequate knowledge of DM complications [9]. A retrospective record review performed in France in 2021 showed that hospitalization for acute diabetic complications decreased in T2D patients after frequent glucose monitoring (-39.4%). In addition, the incidence of diabetic ketoacidosis decreased by more than half in patients with T2D [10]. Furthermore, a cross-sectional study suggested that optimum fasting blood sugar control and diabetes self-management are strongly correlated with a high quality of life in T2D patients with foot ulcers. Therefore, motivating these patients to practice self-management is vital to improve their quality of life and regulate their blood sugar levels [11].

DM has many fatal and life-threatening complications that can be avoided by acquiring sufficient and adequate knowledge about the disease and its complications, having a suitable lifestyle and diet, and most importantly, monitoring the blood glucose level regularly [6]. In Saudi Arabia, studies evaluating the association between glucose monitoring and complications associated with DM are limited. Furthermore, only a few have assessed the effect of glucose monitoring frequency. Hence, this study aimed to assess the effects of regular glucose monitoring on the occurrence of complications in T1D and T2D patients.

## Materials and methods

Study design, setting, and population

This cross-sectional study was performed at King Abdulaziz University Hospital (KAUH) in Jeddah, Saudi Arabia, between June and December 2022. We included both male and female patients diagnosed with T1D and T2D.

Data collection method

A standardized and anonymous questionnaire was distributed electronically using Google Forms. The questionnaire consisted of four sections. The first section acquired demographic information, including sex, year of birth, nationality, marital status, monthly household income, level of education, whether one of the parents is a physician, whether the patient is diagnosed with T1D or T2D, number of years since diagnosis of DM, compliance to diabetic medications, and whether the patient monitors their blood glucose level.

For cases in which the patients monitored their blood glucose levels, the participant was directed to the second section, which queries the patient regarding the frequency of blood glucose level monitoring and how the patient monitors it. If the patient did not monitor their blood glucose level, the participant was forwarded to the third section, which asked why the patient did not monitor their blood glucose levels. The fourth section of the questionnaire was structured and referenced from the related literature [9,12-14]. This section was on the prevalence of complications, such as cardiovascular diseases, hypertension, neuropathy (loss of sensation in the foot or hands), renal diseases, Eye complications (cataract or glaucoma), foot ulcers, and other complications in diabetic patients (Appendices).

Statistical analyses

Microsoft Excel 2019 was used for data entry and statistical analyses were performed using IBM SPSS Statistics version 21 (IBM Corp., Armonk, NY, USA). Frequencies and percentages were calculated for categorical variables, and measures of central tendencies were calculated for continuous variables. Analysis of variance, independent samples t-test, and chi-square test of independence were used. Binary logistic regression was used to adjust the p-values for possible confounders and determine the independently associated factors. CI was set at 95%, and p < 0.05 was considered statistically significant.

Ethical approval

This study was approved by the Biomedical Ethical Committee of KAUH (Ref: 163-22). All participants were notified of the objectives and confidentiality of their responses, and informed written consent was obtained from all participants.

## Results

Participants' social characteristics

This study included 206 diabetic patients with a mean age of 41.2±19.37; out of the selected participants, 113 (54.9%) were females and 93 (45.1%) were males. The demographic information of the participants is presented in Table 1.

**Table 1:** Participants characteristics Note: Values in age are expressed as mean ± standard deviation, and others are expressed as numbers (N) and percentages (%) Abbreviations: N: Number, SD: Standard Deviation

Variables	Mean	SD
Age	41.2	19.37
	N	%
Sex		
Male	93	45.1
Female	113	54.9
Nationality		
Saudi	186	90.3
Non-Saudi	20	9.7
Marital Status		
Single	79	38.3
Married	112	54.4
Other	15	7.3
Highest Level of Education		
Elementary	12	5.8
Intermediate	15	7.3
High school	37	18.0
Diploma	18	8.7
Bachelors	104	50.5
Masters	13	6.3
PhD	7	3.4
Do You Have a Family Physician		
Yes	69	33.5
No	137	66.5
Monthly Household Income		
Less than 3,000 SR	31	15.0
3,001-7,500 SR	33	16.0
7,501-10,000 SR	33	16.0
10,001-15,000 SR	39	18.9
15,001-20,000 SR	24	11.7
More than 20,001 SR	46	22.3

Participants' DM characteristics and glucose monitoring

Among the participants, 110 (53.4%) had T1D and 96 (46.6%) had T2D. Most participants (N=186, 90.3%) took their medications regularly and 188 (91.7%) knew about HbA1C. In addition, 51 (24.8%) patients were known diabetics for more than 15 years ago. Regarding the monitoring of glucose levels, 170 (85.4%) participants monitored their blood glucose levels, with the majority (N=115, 65.3%) monitoring it once or more daily. Of those who monitored their glucose levels, most (73.9%) used at-home blood glucose monitoring devices, 41 (23.3%) used CGM devices, and only five (2.8%) monitored their glucose when visiting the doctor. Table 2 presents information on DM characteristics and glucose monitoring.

**Table 2:** Patients DM characteristics Note: Values are expressed as numbers (N) and percentages (%) Abbreviations:  N: Number.

Variables	N	Percentage
Type of Diabetes		
Type 1	110	53.4
Type 2	96	46.6
Years since diagnosis of diabetes		
<1 Year	32	15.5
1-5 Years	48	23.3
6-10 Years	39	18.9
11-15 Years	36	17.5
>15 Years	51	24.8
Compliance to medications		
Yes	186	90.3
No	20	9.7
Been Hospitalized Before Due to Diabetes		
Yes	74	35.9
No	132	64.1
Do you monitor your glucose level?		
Yes	176	85.4
No	30	14.6
How often do you measure		
Once or more than once a day	115	65.3
Once a week or less	29	16.5
Every three months	5	2.8
Only when I need too	27	15.3
How do you monitor		
At-home blood glucose monitoring	130	73.9
Continuous glucose monitoring	41	23.3
Whenever I visit the doctor	5	2.8

**Figure 1:**
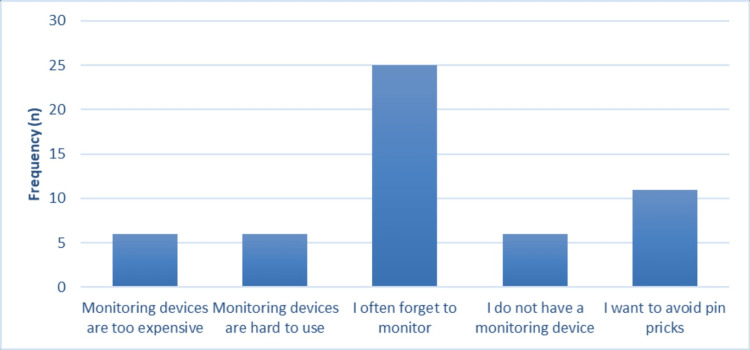
Patients' reasons for not monitoring their blood glucose levels

Regarding the reasons why the participants did not monitor their glucose levels, 25 out of 30 reported that they often forget to monitor and 11 out of 30 (36.7%) did not monitor because they wanted to avoid a pinprick. Figure 1 illustrates the reported reasons for not monitoring glucose levels.

Association between glucose monitoring and DM complications

**Figure 2:**
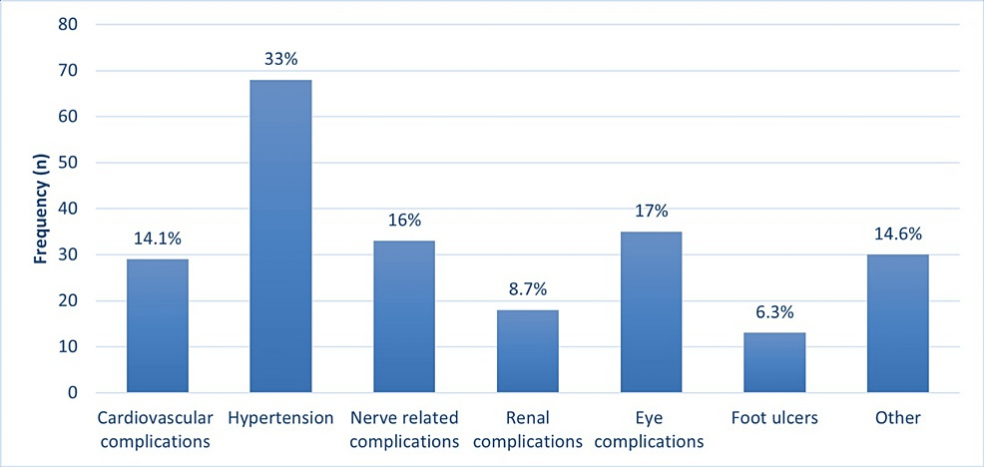
The most frequent DM complications reported

The most common diabetic complications reported by patients were hypertension (n = 68, 33%) and eye complications (n = 35, 17%). Figure 2 illustrates the rest of the participants' complications.

**Figure 3:**
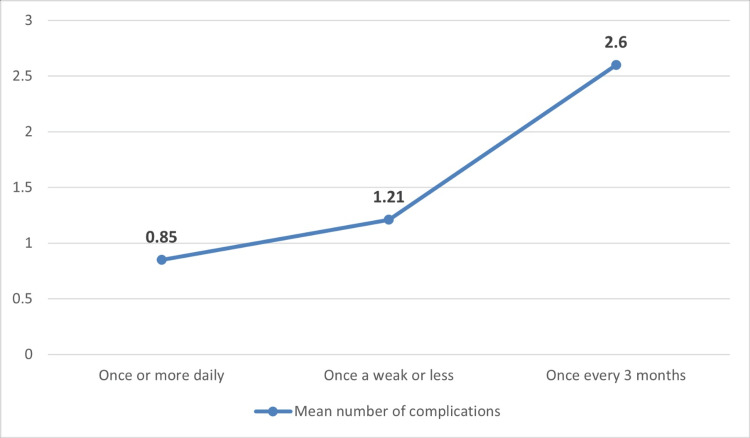
Frequency of glucose monitoring and the mean number of DM-related complications

When assessing the relationship between glucose monitoring and the development of DM complications, the complication rates were found to be similar between those who monitored their glucose levels and those who did not (55.1% vs. 56.7%, p = 1.000). However, frequent monitoring significantly lowered the number of complications, as patients who measured their glucose levels once or more daily had a mean number of complications of 0.85±1.223 compared to 2.60±1.673 in patients who measured once every three months (p = 0.002). Figure 3 illustrates the relationship between the frequency of glucose monitoring and number of DM complications. 

Moreover, CGM was the best monitoring method for lowering the mean number of complications compared to at-home glucose monitoring and monitoring when visiting the doctor, respectively (0.46±0.809 vs. 1.25±1.420 vs. 2.00±2.00, p = 0.002).

DM complication rate had a significant relationship with the patient's age, as the mean age of patients with complications was 50.15±15.331 compared to a mean age of only 30.12±15.331 in patients with no complications (p < 0.001). The results also revealed a significant positive correlation between increased age and number of complications (r = 0.515, p < 0.001). Additionally, patients with T2D had a higher complication rate than those with T1D (58.8% vs. 41.2%; p < 0.001). The relationships between the demographic factors and the rate of DM complications are presented in Table 3.

**Table 3:** Relationships between the demographics and DM characteristics and the occurrence of DM complications Abbreviations: N: number, %: percentage, *: Statistical significance was set at p < 0.05

Variables	N (%)	Rate of DM complications occurrence	P-value
Sex			0.567
Male	93 (45.1)	58.1%	
Female	113 (54.9)	53.1%	
Nationality			0.838
Saudi	186 (90.3)	54.8%	
Non-Saudi	20 (9.7)	60.0%	
Marital status			<0.001*
Single	79 (38.3)	31.6%	
Married	112 (54.4)	72.3%	
Other	15 (7.3)	53.3%	
Monthly household income			0.390
Less than 3,000 SR	31 (15.0)	45.2%	
3,001-7,500 SR	33 (16.0)	45.5%	
7,501-10,000 SR	33 (16.0)	54.5%	
10,001-15,000 SR	39 (18.9)	66.7%	
15,001-20,000 SR	24 (11.7)	54.2%	
More than 20,001 SR	46 (22.3)	60.9%	
Age group			<0.001*
<19	28 (13.6)	14.3%	
20-39	70 (34.0)	38.6%	
40-59	63 (30.6)	66.7%	
60-79	40 (19.4)	95.0%	
>80	3 (1.5)	66.7%	
Highest level of education			0.009*
Elementary	12 (5.8)	41.7%	
Intermediate	15 (7.3)	40.0%	
High school	37 (18)	43.2%	
Diploma	18 (8.7)	38.9%	
Bachelors	104 (50.5)	59.6%	
Masters	13 (6.3)	92.3%	
PhD	7 (3.4)	85.7%	
Have a family physician			0.014*
Yes	69 (33.5)	68.1%	
No	137 (66.5)	48.9%	
Type of diabetes			0.001*
Type 1	110 (53.4)	42.7%	
Type 2	96 (46.6)	69.8%	
Duration since diagnosis with DM			0.009*
<1 Year	32 (15.5)	34.4%	
1-5 Years	48 (23.3)	43.8%	
6-10 Years	39 (18.9)	61.5%	
11-15 Years	36 (17.5)	66.7%	
>15 Years	51 (24.8)	66.7%	
Take medications regularly			0.458
Yes	186 (90.3)	56.5%	
No	20 (9.7)	45.0%	

A multivariate regression analysis was done to account for all possible confounders that could affect the occurrence of diabetic complications, including sex, age, nationality, marital status, household income, level of education, type of DM, duration since diagnosis, Adherence to medication, prior hospitalization, monitoring of blood glucose levels, frequency of monitoring, and the type of monitor used. Advancement in age was shown to be the only factor that was independently associated with a higher rate of DM complications occurrence (p = 0.002, OR = 1.069, 95% CI = 1.025-1.116).

## Discussion

This study determined the relationship between DM complications and the frequency of glucose monitoring in patients with diabetes in Saudi Arabia. This study showed that the rate of DM complications was similar between patients who monitored their glucose levels and those who did not. However, when assessing the relationship between the frequency of monitoring and the number of complications, there was a significant decrease in the mean number of complications in patients who monitored their glucose frequently. These results are consistent with those of a study performed in the United States that showed that patients who used SMBG devices more regularly had significantly lower HbA1c levels than those who did not [15]. The possible reason could be that patients who frequently monitor their glucose levels maintain lower HbA1c levels, leading to a lower risk of developing complications. Additionally, HbA1c is independently associated with a high risk of heart disease and stroke [16].

This study also found that the patients who used CGM devices had a lower mean number of complications than those who used other monitoring methods. Similar results were reported in two previous meta-analyses, in which CGM was shown to be the best glucose monitoring method for lowering HbA1c levels [17,18]. The benefit of using CGM could be due to the fact that glucose can be monitored regularly, ensuring a high monitoring frequency, which as mentioned, results in better outcomes.

Our results showed that the top reason for patients not to monitor their blood glucose levels was that they often forgot to or that they wanted to avoid pinpricks. Similar results were demonstrated in previous studies, where these two independent factors may affect the decision of monitoring or its frequency among patients with DM [19,20]. Using CGM devices could be a solution to this problem, as a single sensor could be inserted for several days [21]. This will help decrease the number of pinpricks that the patient should go through while also helping to eliminate the forgetfulness aspect of the problem.

The most common complication in our study population was hypertension, and the mean age of patients was 41.2±19.37. One explanation for hypertension being the most common complication could be that the patients had the two most common risk factors for hypertension: DM and aging. However, hypertension may have been associated with DM rather than being a consequence of it. In a previous study, this was explained by the fact that both diabetes and hypertension are the end results of metabolic syndrome, which can develop in the same person and follow similar pathways [22].

A significant relationship between the rate of diabetic complications and the advancement of age was also observed after logistic regression, as the advancement of age was independently associated with higher rates of complications. Similarly, a study performed in the United States revealed that mortality, cardiovascular problems, and hypoglycemia rates all increased sharply with age [23]. Therefore, this prompts that diabetic patients, especially those advanced in age, should be regularly screened for diabetic complications in order to avoid their development.

Strengths and limitations

To the best of our knowledge, this is the first study to explore the effects of glucose monitoring on the rate of DM complications in a Saudi Arabian community, and this study did not only demonstrate the effect of glucose monitoring but also demonstrated the reasons for patients not to monitor. However, there were some limitations. Due to the cross-sectional design of this study and the retrospective nature of the questions, recall bias may have affected the results. Furthermore, some data was missing, as two patients did not fill in their ages in the questionnaire. Moreover, certain variables such as the history of smoking, alcohol intake, and type of medication were not inquired about in the questionnaire.

## Conclusions

This study established a significant relationship between the frequency of glucose monitoring and the number of DM complications. We also found that CGM was the best monitoring method because it was associated with the lowest number of complications. In addition, the most common reason for not monitoring glucose levels was not remembering to do so. Therefore, we recommend that physicians encourage diabetic patients to begin using CGM as it helps increase the frequency of monitoring while eliminating the human error of forgetting to monitor. We also recommend that future clinical trials in Saudi Arabia with a control group and a larger sample size to be conducted for an improved understanding of the relationship between the frequency of glucose monitoring and the number of DM complications.

## Appendices

Research questionnaire

The aim of this study was to assess the effect of regular glucose monitoring on the occurrence of diabetes complications. All information collected in this survey will be kept strictly confidential and used only for research purposes.

Section 1: Demographic data

Sex:

1-     Male

2-     Female

Year of birth: .....

Nationality:

1-     Saudi

2-     Non-Saudi

Marital Status:

1-     Single

2-     Married

3-     Other

Monthly household income:

1-     Less than 3,000 Saudi Riyals

2-     3,001-7,500 Saudi Riyals

3-     7,501-10,000 Saudi Riyals

4-     10,001-15,000 Saudi Riyals

5-     15,001-20,000 Saudi Riyals

6-     More than 20,000 Saudi Riyals

Level of education:

1-     Elementary

2-     Intermediate

3-     High school

4-     Diploma

5-     Bachelors

6-     Masters

7-     PhD

Is one of your family members a physician?

1-     Yes

2-     No

Are you a type 1 or type 2 diabetic?

1-     Type 1

2-     Type 2

How long has it been since you have been diagnosed with diabetes?

1-     Less than 1 year

2-     From 1-5 years

3-     From 6-10 years

4-     From 11-15 years

5-     More than 15 years

Do you take your diabetes medications regularly?

1-     Yes

2-     No

Have you ever been hospitalized before due to diabetes?

1-     Yes

2-     No

Do you know what cumulative blood sugar (HbA1c) is?

1-     Yes

2-     No

How often should you measure your cumulative blood sugar (HbA1c)?

1-     Every month

2-     Every 3 months

3-     Every 6 months

4-     Every year

Do you monitor your glucose level?

1-     Yes

2-     No

Section 2: Blood glucose monitoring

(This section was directed to those who monitor their blood glucose levels)

How often do you measure your glucose level?

1-     Once or more than once a day

2-     Once a week or less

3-     Every 3 months

4-     Only when I need to

How do you monitor your blood glucose level?

1-     I use at home blood glucose monitor

2-     I use a continuous glucose monitoring device

3-     Whenever I visit my doctor

4-     Other

Section 3: Reasons for not monitoring blood glucose

(This section was directed to those who do not monitor their blood glucose levels)

Why don't you monitor your glucose levels?

1-     Cost

2-     It is too hard to use

3-     I often forget to monitor

4-     I do not have a monitoring device

5-     I want to avoid using a pin prick

Section 4: Complications

(This section is aimed to help us record the rate of complications)

Have you experienced any of these problems before?

Cardiovascular diseases:

1-     Yes

2-     No

Hypertension:

1-     Yes

2-     No

Nerve damage (loss of sensation in feet or hands):

1-     Yes

2-     No

Renal disease:

1-     Yes

2-     No

Eye complications (Cataract, Glaucoma):

1-     Yes

2-     No

Foot ulcers:

1-     Yes

2-     No

Other:

1-     Yes

2-     No
